# Time Multiplexed Active Neural Probe with 1356 Parallel Recording Sites

**DOI:** 10.3390/s17102388

**Published:** 2017-10-19

**Authors:** Bogdan C. Raducanu, Refet F. Yazicioglu, Carolina M. Lopez, Marco Ballini, Jan Putzeys, Shiwei Wang, Alexandru Andrei, Veronique Rochus, Marleen Welkenhuysen, Nick van Helleputte, Silke Musa, Robert Puers, Fabian Kloosterman, Chris Van Hoof, Richárd Fiáth, István Ulbert, Srinjoy Mitra

**Affiliations:** 1 Imec, 3001 Leuven, Belgium; firatyazicioglu@gmail.com (R.F.Y.); moralope@imec.be (C.M.L.); Marco.Ballini@imec.be (M.B.); Jan.Putzeys@imec.be (J.P.); Shiwei.Wang@imec.be (S.W.); Alexandru.Andrei@imec.be (A.A.); Veronique.Rochus@imec.be (V.R.); Marleen.Welkenhuysen@imec.be (M.W.); Nick.VanHelleputte@imec.be (N.v.H.); Silke.Musa@imec.be (S.M.); robert.puers@esat.kuleuven.be (R.P.); Chris.VanHoof@imec.be (C.v.H.); 2 Electrical Engineering Department-ESAT, KU Leuven, 3001 Leuven, Belgium; 3 Faculty of Psychology and Educational Sciences, KU Leuven, 3000 Leuven, Belgium; Fabian.Kloosterman@nerf.be; 4 Neuro-electronics Research Flanders, 3001 Leuven, Belgium; 5 VIB, 3000 Leuven, Belgium; 6 Institute of Cognitive Neuroscience and Psychology, Research Centre for Natural Sciences, Hungarian Academy of Sciences, H-1117 Budapest, Hungary; fiath.richard@ttk.mta.hu (R.F.); ulbert.istvan@ttk.mta.hu (I.U.); 7 Faculty of Information Technology and Bionics, Pázmány Péter Catholic University, H-1083 Budapest, Hungary; 8 School of Engineering, University of Glasgow, Glasgow G10 8QQ, UK; srinjoy.mitra@glasgow.ac.uk

**Keywords:** active electrode, active neural probes, CMOS, high density component, neural amplifier, neural array, neural recording

## Abstract

We present a high electrode density and high channel count CMOS (complementary metal-oxide-semiconductor) active neural probe containing 1344 neuron sized recording pixels (20 µm × 20 µm) and 12 reference pixels (20 µm × 80 µm), densely packed on a 50 µm thick, 100 µm wide, and 8 mm long shank. The active electrodes or pixels consist of dedicated in-situ circuits for signal source amplification, which are directly located under each electrode. The probe supports the simultaneous recording of all 1356 electrodes with sufficient signal to noise ratio for typical neuroscience applications. For enhanced performance, further noise reduction can be achieved while using half of the electrodes (678). Both of these numbers considerably surpass the state-of-the art active neural probes in both electrode count and number of recording channels. The measured input referred noise in the action potential band is 12.4 µVrms, while using 678 electrodes, with just 3 µW power dissipation per pixel and 45 µW per read-out channel (including data transmission).

## 1. Introduction 

**Figure 1:**
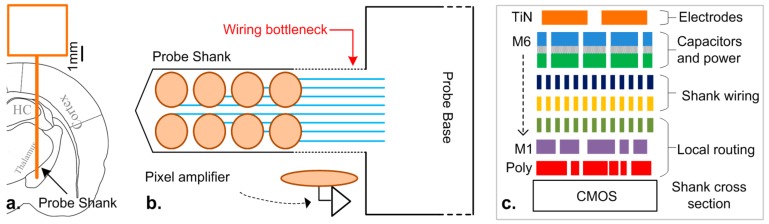
(**a**) Schematic of implanted probe reaching multiple areas inside a rat brain; (**b**) neural probe with shank wiring bottleneck limiting the number of electrodes; (**c**) typical CMOS (complementary metal-oxide-semiconductor) back end-of-line cross section, with six metal layers.

The need for large-scale neural recording across multiple brain areas in behaving animals has driven the recent development of high density neural probes [1]. In this application, the implanted probe shank needs to be sufficiently long to reach deep brain structures (Figure 1a), but it also needs to have a reduced cross section to minimize tissue damage. Active silicon neural probes that have been recently developed consist of a large number of tiny active electrodes that can locally amplify/buffer the neural signals [1,2,3,4]. However, with such limited space for each active electrode, the CMOS (complementary metal-oxide-semiconductor) pixel amplifiers (PA) underneath the electrodes are restricted to a bare minimum, while most of the signal processing is done in the ‘base’ (i.e., non-implantable part) of the probe.

This manuscript presents a thorough description and in vivo measurements from a probe architecture first published in [5], presenting an active neural probe that contains 1344 recording (20 µm × 20 µm) pixels and 12 reference pixels (20 µm × 80 µm), densely packed on a 50 µm thick, 100 µm wide and 8 mm long shank. This new type of probe features a 1:1 electrode-to-channel ratio and supports simultaneous recording of all of the 1356 electrodes (full-probe recording) and high-performance recording from 678 electrodes (half-probe recording), increasing the number of simultaneous recording channels by 3.5 times when compared to the state of the art [4]. Each active electrode (i.e., pixel) consists of dedicated in-situ circuits for signal source amplification that are located under each electrode. 

Dedicated neural amplifier circuits [6,7,8] can provide the best electrical performance, however, they need to be connected to external passive probes (e.g., [9]) or arrays (e.g., [10]) that capture the signal. Such an arrangement is scalable to only tens or hundreds of channels [11], due to the limitation in the interconnection between the external probe and the amplifier circuit. Furthermore, this leads to an overall less compact system, which is where CMOS neural probes present an advantage, as they can integrate both of the electrodes and circuits in a single integrated circuit [12].

**Figure 2:**
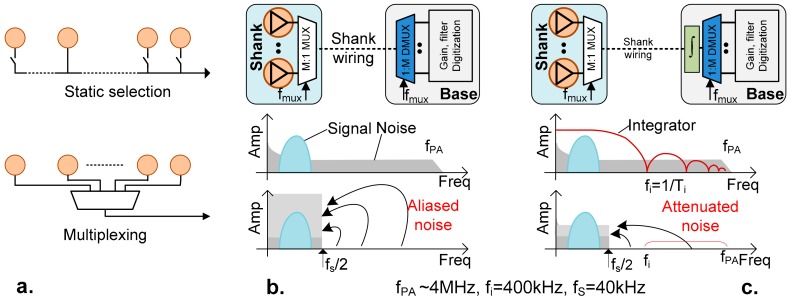
(**a**) Traditional approach (**top**) employs a static switch which allows only a single active electrode to be read at the same time, while the new approach (**bottom**) allows all of the active electrodes to be readout through multiplexing; (**b**) consequences of multiplexing without filtering; (**c**) filtering signal by integration reduces out-of-band thermal noise.

Prior active [2,3,4] and passive [9,13,14,15] neural probes used a dedicated metal line per electrode to send the signal to the base circuitry. This one-to-one mapping results in either a limited number of electrodes present on the shank [9] or the recording of a statically selected subset [2,4,15] (Figure 2a). Naturally, these approaches limit the number of simultaneous recording electrodes to the number of metal lines fitted in the cross section of the shank (Figure 1b). The available routing space is shared amongst signal wires, local routing, power, and input coupling capacitors (Figure 1c). Smaller CMOS processing nodes may alleviate this problem by allowing a higher routing density, however this approach comes with the drawback of increased crosstalk amongst channels [12]. Further degradation of the signal will be caused by the increased thermal noise caused by the increased electrical resistance. When the wire connecting the electrode to the base reaches magnitudes of tens of kΩ or more, the overall contribution can become significant. Therefore, a smaller CMOS node may not be the solution to an increased number of simultaneous read electrodes. 

To overcome the fundamental wiring bottleneck and achieve a denser simultaneous readout, a new architecture is proposed, which relies on time division multiplexing and techniques that reduce the associated drawbacks of implementing multiple sensitive and low noise switched circuits across a long and narrow shank. The shank imposes strict area and power limitations, resulting in a poor power supply with increased drop and ripple, dense layout prone to capacitive coupling, and a requirement for low complexity circuits, which provide the desired functionality and low noise. 

This architecture and circuit implementations presented maximize the readout capability of a given inserted shank by simultaneously recording all of the available electrodes. Thus, a probe fully covered with electrodes, which are all simultaneously readable, will provide the neuroscientist with the maximum amount of information for the damage created by the probe insertion. This aspect is a crucial drive for further development, as current neural recordings are done at a scale that is minute when compared to the size of a brain. Furthermore, the high density was shown to improve the performance of spike sorting [16]. This new architecture further opens new ways in the scaling of neural probes by circumventing the barrier of a limited number of shank wires. 

The paper is organized as follows: in Section 2 the operation principles of the probe are described in the context of our goal. Section 3 describes the overall architecture and functionality, continuing with further details of specific novelty circuit blocks described in Section 4. In Section 5 we describe the resulting fabricated device, following with the details of the supporting system blocks required to use the probe in Section 6. Test results with both electrical and in vivo measurement are outlined in Section 7, reaching to conclusions in Section 8.

## 2. Operation Principles

### 2.1. Overview

Active neural probes improve recording quality when compared to the passive versions by buffering or amplifying the input signal close to its source (i.e., the electrode). This approach reduces the source impedance and minimizes the crosstalk caused by the coupling amongst the long and dense neighboring shank wires [2]. In such cases, the electronics under each active electrode (i.e., PA) has strict design constrains. 

Within a shank that is fully covered by electrodes, the area available for each amplifier is limited by the electrode size, the power is limited by the acceptable tissue heating, and the noise requirements are imposed by the signal amplitude (as small as tens of µV). 

In previously active and passive probes, only a fraction of the electrodes present may be read out simultaneously [2,4,15]. Static switches need to be configured before recording (Figure 2a, top), as the amplifiers used have a long settling time required in order to capture neural signals down to <1 Hz, while still rejecting the DC offset of the electrode. This configuration allows for a certain degree of flexibility in choosing which probe area is read out, however the approach is limiting, as it does not give neuroscientists the opportunity of accessing all of the brain areas near the probe simultaneously. Overcoming this limitation is achieved by employing a multiplexing architecture which makes use of new types of amplifiers, capable of operating in such a multiplexed configuration (Figure 2a, bottom), while still maintaining the stability and performance required to record the neural signals.

### 2.2. Noise Folding

Within the limited pixel area, an obvious method to reduce noise is to increase the current consumption of the PA input transistor. This results in the PA having a high bandwidth. Since the neural signal band itself is limited to ~7.5 kHz, the PA output can be sampled at a frequency *f_s_* > 15 kHz in the base. Therefore, a simple time division multiplexing could be embedded within the shank (Figure 2a), allowing M number of PA outputs on a single shank wire (using a sampling frequency *f_MUX_ = M × f_S_*). 

However, the lack of a traditional anti-aliasing filter limiting the high PA bandwidth increases the in-band noise (coming from both brain and circuits) due to spectral folding. Since it is not possible to fit low pass filters within the limited area of the PA (before the sampling operation), we have employed an alternative method of noise reduction by integrating the signal over a period of time (*T_i_*) (Figure 2c) [17]. Since the integration circuits are located after the sampling circuits, they may be placed within the base, not the area restricted pixels. The integrate, sample, and reset operations strongly attenuate the signal beyond *f_i_* = 1/*T_i_* (*f_i_ ≥ f_MUX_*), improving the signal-to-noise ratio while allowing for certain circuit elements to be shared across multiple channels.

For the current probe design, a multiplexing factor of *M* = 8 was sufficient to overcome the shank-wire bottleneck and provide sufficient area for power lines and capacitors. To avoid in-band distortion, each channel is oversampled at *f_s_* = 40 kHz (higher than the Nyquist rate of 15 kHz), producing a total multiplexing frequency of 320 kHz. This, in turn, limits the integration period to a maximum of 3.125 µs. We have used *T_i_* = 2.5 µs, while using the remaining time for circuit transitions between the adjacent channels that were selected for multiplexing. This process effectively results in a low pass operation, strongly reducing the PA bandwidth from ~4 MHz to 400 kHz and limiting the noise folding [18].

### 2.3. Power Limitation

One of the most stringent restrictions of an implantable device is on power dissipation and the resulting heating of the nearby biological tissue. In this application, the power budget of the probe is determined by the limited amount of heat that may be dissipated without disturbing or damaging the surrounding brain tissue. For long-term experiments with a chronically implanted probe in the brain, a maximum of 1 °C [19] is considered acceptable, while for acute or shorter term recordings a higher temperature increase may be acceptable. Using finite element method (FEM) simulations with Comsol Multiphysics^®^, we determined the maximum power that the circuit can dissipate, prior to its design. Thus, we have determined the power budget such that the hottest point in the brain is seeing at most 1 °C temperature increase. A similar approach was used in previous designs [2,4], which have already been used in long term recordings. 

**Figure 3:**
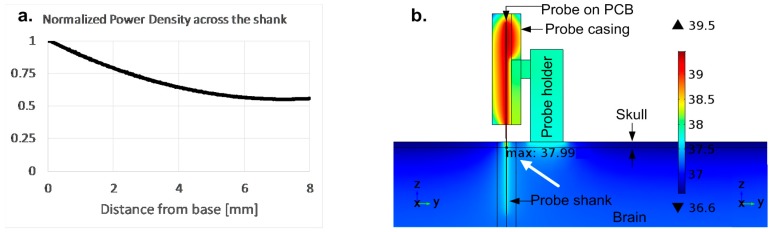
(**a**) Normalized power density across the shank due to increasing current and supply towards the base; (**b**) thermal simulations of probe with holder implanted in the brain, showing maximum temperature of 38 °C reached at the edge of the brain. This simulation was used to determine the maximum safe power that may be dissipated in the shank.

The critical part, the implanted shank, is modeled taking into account the variation of the power dissipation across its area, while the base is modeled with uniform power density (Figure 3a). Furthermore, the complete probe as well as its fixture are modeled along with the skull, dura, and brain, including blood circulation. A model is used to determine the non-uniform power distribution across the shank, taking into account the dissipation of the amplifiers and power lines according to the layout (Figure 3a). The power dissipated in the power lines brings a significant contribution nearing the base. As shown in Figure 3b, the maximum temperature is reached at the edge between the brain and skull, as this area of the shank has the highest power density caused by the highest value of supply voltage as well as the highest current in the supply rail, taking into account the worst-case scenario that is expected from the probe. 

According to the simulation results, the power dissipation limits that would produce a 1 °C increase in the tissue temperature are 4.5 mW for the entire implanted shank and 45 mW for the base. These determined limits were used as design specifications for the circuits: the complete power budget is used in the shank when all of the electrodes are turned ON in order to minimize noise, while the base circuits require less power than allowed, without a penalty on performance. Thus, the design presented further increases the electrode density within the available power and noise limits.

## 3. Architecture

Taking into account the previously described operating principles, we propose a neural probe architecture, which is described in detail in this and the following sections. 

**Figure 4:**
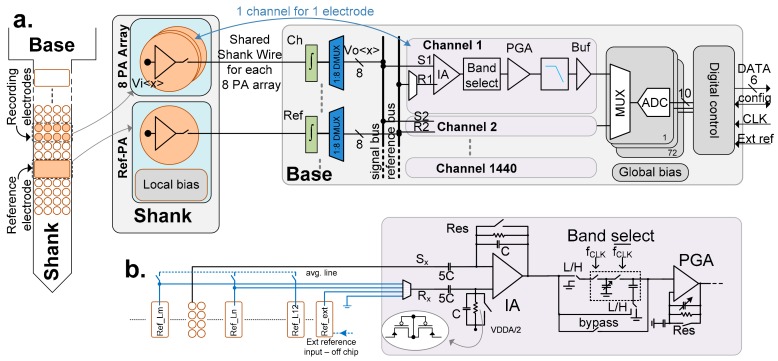
(**a**) Probe architecture showing the device contains one channel for each electrode; left: the recording and reference electrodes on the probe and their corresponding signal blocks; (**b**) Channel details showing (symbolically) the electrode connections and reference selection options available on each channel (2 local, external or ground); an averaging line is used to connect any of the 12 local reference together and average the signal across them; the amplification section consists of AC (alternating current) coupled instrumentation amplifier (IA) and programmable gain amplifier (PGA), along with configurable band selection and cutoff corner filter.

Figure 4a shows the block level architecture of the complete probe, including the number of repeated instances for the relevant parts. In an array of eight PAs, the input signals (*Vi<1:8>*) are connected to each of them individually. The multiplexed output from this array is sent to the base through a shared shank wire. The signal is subsequently fed to an integrator the output of which is demultiplexed (DMUX block) using eight sample-and-hold circuits (*Vo<1:8>*). Each Vo signal then goes to its corresponding channel block (Figure 4b) where the signal is further amplified and filtered, keeping only the band of interest. Together, these blocks implement the noise reduction technique described in Section 2.2, with individual blocks further described in Section 4. 

The outputs of 20 channels are multiplexed and digitized with the help of a 10-bit successive approximation register (SAR) analog to digital converter (ADC) [4]. The number of multiplexed channels is selected based on the required sample rate per channel (20 kHz) and the performance of the selected ADC architecture. 

The digital control block is responsible for generating the internal clocks for the ADCs and the MUX/DMUX blocks from a single external clock source. It also buffers and then serializes the parallel data from all of the ADCs to only six data lines. The number of data lines is a compromise between fewer output lines and lower clock speed (i.e., lower I/O pads dissipation which are part of the base). All of the channels, PAs and bias parameters are configurable through daisy-chained shift registers. 

The chip contains 1344 small electrodes (20 µm × 20 µm) and 12 larger electrodes (40 µm × 80 µm) that can be used as reference. The shank is divided in 12 identical regions, each with a reference electrode in the center and 112 small electrodes around it. The pitch of the small electrodes is given by the compromise between desired number of recording sites at high density and the signal quality that can be achieved with the available area and power budget. Similar to the internal shank reference electrodes, a 13th Ref-PA block without an exposed electrode contact is used to amplify an external reference signal provided through a bond pad. This block is placed at the beginning of the shank, near the base to improve matching to other reference signal amplifiers. 

A total of 180 Integrator-DMUX blocks drive the 1440 channels (1:8 ratio), which are digitized by 72 ADCs (1 ADC per 20 channels). The extra channels (1357 and higher) are used for the external reference and for test purposes.

A global bias block contains a band-gap reference and the necessary circuits to generate the required voltages and currents for the chip. Hierarchical and active biasing is used to facilitate the biasing of such a high number of analog blocks spread across the whole base area.

## 4. Circuit Description

### 4.1. Pixel

**Figure 5:**
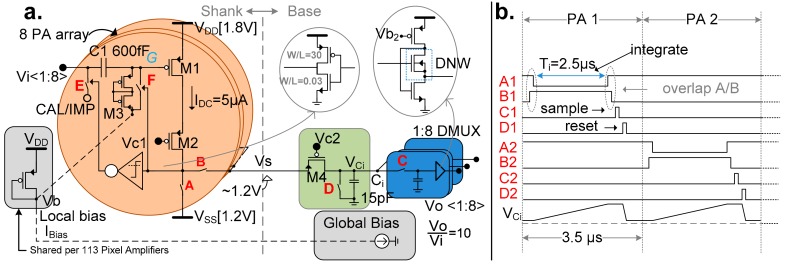
(**a**) Pixel amplifiers (PA) architecture: M1 works as gm stage. The cascode transistor M2 isolates M1 from the clock feedthrough at the output and overlapped A/B switches enable M1 to always have an ON current. Both these methods along with proper layout placement ensure the stability of high impedance node G. The S/H circuit uses flipped voltage follower buffer with a deep N-well NMOS; (**b**) timing diagram showing the switching cycles of 2 consecutive PAs.

The integrator architecture, described in Section 2.2, is split in two parts. Within the limited area of the pixel, the PA acts as a voltage to current converter (Figure 5a), while the integration capacitor and sample and hold (S/H) circuits forming the de-multiplexer are located in the less area-restricted base. The current from the pixels is first integrated for a fixed period of time (*T_i_* = 2.5 µs) over a capacitor (*C_i_* = 15 pF) that is shared by eight channels. After *T_i_*, the voltage on *C_i_* is sampled and then the capacitor is discharged for the next cycle (Figure 5b). The S/H circuit is followed by a buffer, implemented as a flipped voltage follower [20] and using a deep N-well NMOS transistor. The buffer is necessary for the reference path (Ref DMUX) where one output may connect to multiple channels (Figure 4a). In the signal path, it is primarily used to closely match the reference path. 

The PA employs an open-loop, AC-coupled, transconductance (*gm*) stage (*M1*). At the end of the DMUX, this produces an overall small signal gain of 10, given by:

(1) $$A=\frac{v_{o}}{v_{i}}=gm\frac{T_{i}}{C_{i}}$$

The cascode transistor, *M2*, reduces the clock feedthrough from the switches *A* and *B* to the *gm* stage. These switches are operated with temporal overlapping to ensure a constant ON current through *M1*. These aspects are crucial to maintaining DC operating point stability, as the gate (*G*) of *M1* is a high impedance node (~TΩ), produced by the high-pass filter. The filter (corner << 1 Hz) is necessary to reject the relatively high input DC level (upwards to hundreds of mV) produced by the electrode-tissue interface [21], while allowing through neural signals down to 1 Hz. Due to the small value of *C1*, the two transistors forming the pseudoresistor (*M3*) are considerably long, taking up a significant area in the pixel. 

During normal operation, the cascode transistor *M4* located between the current source (i.e., the PA) and the integrating capacitor (*C_i_*) ensures that the shank wire connected at the source of *M4* is at a constant voltage equal to the supply rail (*V_s_* ~ 1.2 V). By keeping all of the shank wires at a constant voltage, this approach reduces the crosstalk amongst channels caused by the capacitive coupling of the long shank lines. Furthermore, the shank power dissipation is reduced, as this constant voltage is higher than the average voltage on the top plate of the integrating capacitor, *C_i_*, causing an overall smaller average *V_DS_* across *M2*. 

The layout of the pixel is designed to take certain aspects into account, besides the stringent size restrictions. The pixels are isolated from each other through a dedicated guard ring. Furthermore, the routing within the pixel is such that *M3*, *M1* and *M2* are properly shielded from external disturbances caused by the switching elements, *A* and *B*, as well as the digital control lines. 

With the exception of the high-threshold inverter used for calibration (Section 4.3), the transistors, shown in Figure 5, are thick-oxide transistors, in order to reduce gate leakage and facilitate operation at higher supply levels (i.e., 1.8 V).

### 4.2. Shank Power Supply

The choice of voltage levels for the supply rails of the PA is defined by multiple factors. The power budget (*I_DC_* × *(V_DD_* − *V_SS_*)) determined in Section 2.3, coupled with minimal noise requirement, induces a trade-off between the current through *M1* and *M2,* and their V_DS_. However, the chosen operating point must account for the drop in the power supply lines across the shank. A 0.6 V supply voltage was found to be optimal. By using *V_SS_* = 1.2 V and *V_DD_* = 1.8 V, the current can be directly integrated over *C_i_* (within the range of 0 to 1.2 V), eliminating the need for a negative supply or mirroring circuitry. Furthermore, since the 1.2 V rail is used by the following stages, the current from the unselected pixels (switch *A* closed) can be fed back into the 1.2 V rails of other blocks in the base, reducing the overall consumption of the probe. 

**Figure 6:**
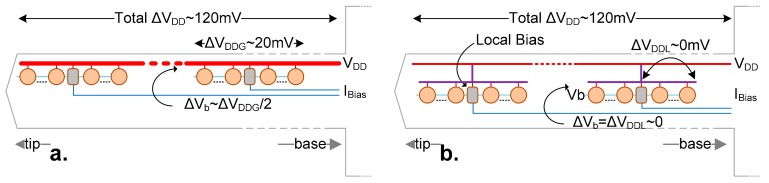
(**a**) Large supply drop across the 8 mm long shank changes the bias voltage, ΔV_b_. This is due to the high current in the supply rail (consumed by all PAs), that causes voltage drop within a bias region (ΔV_DDG_); (**b**) a tree-like power supply ensures that supply change ΔV_DL_ is close to zero within each region, due to the lower current in the local rail. Each region contains its dedicated local bias generator.

The extremely high aspect ratio of the shank (80:1), along with the limited area for supply routing (due to the large number of signal wires), results in a high voltage drop of ~120 mV across each of the shank power supply lines (Figure 6a). Two complimentary solutions mitigate the negative consequences of the voltage drop. First, the gate bias, *V_b_*, is generated locally and periodically across the shank using reference currents from the base, as the limited space does not allow for a more complex solution that is sufficiently accurate. There are 12 bias circuits, one for each of the 12 regions of the probe shank. Still, the voltage drop experienced within the same bias group creates sufficient differences among the PA bias voltages (Δ*V_b_* ~ Δ*V_DDG_*/2) to affect the operation performance. To further mitigate this issue, a tree structure for the supply line is implemented by splitting the shank pixel amplifiers in branches, one for each of the 12 regions (serving 113 PAs each), each powered from the same bus through a single connection. Here, each half of a branch experiences an insignificant supply drop (Δ*V_b_* ~ 0 V), due to a much lower consumption (i.e., only 56 pixels). This results in a more controlled bias current amongst different pixels in a group. 

The power rails of the shank are carried over the top metal layers, 5 and 6 as shown in Figure 1c. A minimal amount of power supply decoupling is provided by using the two power rails to form the two layers of a metal-insulator-metal (MIM) capacitor, which is distributed across the shank (total ~80 pF) and does not consume extra area. The input capacitor *C1* of the PA (Figure 5) is also found implemented on the top two metal layers, which results in a tradeoff between the area used for power rails and input capacitance, which influences the noise performance. More decoupling capacitors for the shank power supply are present in the base and shank neck, as well as external components on the outside of the probe, in proximity to the power pins.

### 4.3. Calibration and Reset

The pixel circuits offer additional features that can be activated independently per pixel, one at a time, without containing a dedicated memory element such as shift registers.

Both gain calibration (CAL) and electrode impedance characterization (IMP) are activated through switch *E* in the pixel (Figure 5a). By applying a known voltage (via the CAL/IMP port) while the electrode is floating (not connected to sample or solution, e.g., before implantation) the end-to-end gain can be measured and calibrated. Similarly, the electrode-tissue interface impedance can be characterized by applying a known current from the circuit side, while the probe is submerged in a grounded saline solution, by measuring the voltage that develops at the pixel input. Since this measurement requires the connection of a single PA input to the shared CAL/IMP signal, the selection of the corresponding switch *E* is done by temporarily lowering the wire voltage *Vs* to ~0.8 V by controlling cascode voltage *Vc2* when its switch *B* is ON. This triggers a high-threshold inverter only within the selected PA, thus setting the switch *E*, while allowing normal operation of the PA, albeit with a higher power dissipation due to a higher *V_DS_*. This method of using the output line simultaneously as a select signal eliminates the need for dedicated registers within the PA, which take valuable area. The signals required for calibration are generated externally by the headstage, as described in Section 6. 

The low frequency high pass corner (<1 Hz) formed by the AC coupling filter leads to significant settling time at startup or in response to large voltages induced by nearby brain stimulation. To reduce the time needed to reach a steady state, the filter resistor (*M3*) can be shorted using switch *F*, resulting in a settling time in the order of micro seconds. Furthermore, this can be used in conjunction with optical stimulation, as the pseudoresistor is a light sensitive structure. By preemptively activating the reset before the light pulse and releasing it after, the pixel may avoid being affected. Similar to switch *E*, this switch is controlled by the voltage present on the output line. Specifically, the switch is closed and a PA reset is triggered by a logic low level (<0.6 V) achieved by controlling the wire voltage Vs. 

### 4.4. Recording Performance

Although small scale designs have been proposed [22], multiplexing fast enough to capture the full signal band on the shank has not been previously demonstrated on a large scale as it poses a multitude of challenges. Due to the switching nature of the circuits and in order to maintain proper operation under large voltage drops while also accounting for supply ripple on the highly resistive power lines, any 6 of the 12 shank regions can be turned ON simultaneously without an additional penalty on noise and power dissipation (half-probe recording). This permits recording with good noise performance from six arbitrary regions on the 8-mm shank (~0.7 mm each, covering 4 mm), which is sufficient for covering multiple regions of a rat brain. 

Moreover, the design also supports the simultaneous readout from all of the electrodes on the shank (1356) by featuring 1440 channels in the base (full-probe recording). This recording scenario comes with increased noise due to the very small amount of decoupling capacitors in the shank, as insufficient area is available to properly filter the power rails at a higher current consumption. However, as illustrated in Section 7.2, these recordings still provide sufficient signal to noise ratio (SNR) for accurate analysis of the data. 

### 4.5. Channel

Each channel receives a signal (Sx) and reference (Rx) line, from the corresponding DMUX (Figure 4a) that feeds the instrumentation amplifier (IA). The referencing and differential amplification allows for improvement of the common mode rejection ratio (CMRR). The reference (REF) line can be selected from (i) one of the local reference PA (Ref-PA), (ii) a few locally averaged Ref-PAs, or (iii) an external signal. The various reference signals facilitate the recording of different brain signals: action potentials (AP) and local field potential (LFP) have different spatial resolution and may benefit from a local or global reference (e.g., a screw attached to the animal skull), depending on specific recording conditions [23]. Furthermore, single ended operation is possible, which along with the readout of the reference channels, enables software referencing, potentially resulting in improved signal quality [23].

In order to preserve circuit symmetry and avoid distortions, each Ref-PA is de-multiplexed to eight outputs, such that for each channel the two inputs of the IA are de-multiplexed (i.e., sampled) simultaneously.

By providing a gain of 10, the integrator also relaxes the noise budget of the IA. The IA is implemented using an AC-coupled folded-cascode operational transconductance amplifier (OTA), with the bandwidth being limited to ~15 kHz. This prevents aliasing from the subsequent switched capacitor (SC) band-select filter.

The SC filter is implemented as a first order RC-filter and operates at 80 kHz. Through a selection of switches (Figure 4b), it can be configured as high pass, low pass, or disabled. Furthermore, the corner can be programmed through the change in capacitance value to either 300, 500 or 1000 Hz. This allows a selection of the action potential band (AP: 300/500/1000 Hz to 7.5 kHz), the local field potential band (LFP: <1 Hz to 300/500/1000 Hz) or the full band (<1 Hz to 7.5 kHz), respectively, by bypassing the filter.

A programmable gain amplifier (PGA) follows the filter and provides eight configurable gains between 1 and 50. The PGA is DC coupled to the previous stage and uses a capacitive feedback to provide the variable AC gain, while the DC gain is 1. The role of the PGA is to maximize the utilization of the dynamic range of the ADC, since neural signals will vary in amplitude based on the selected band and brain region. After the PGA, the signal passes through an anti-aliasing filter and is buffered, prior to being multiplexed and fed into the ADC. A class-AB ADC driver is used to reduce the static power consumption. 

Each channel allows for independent band selection, gain configuration, reference selection, calibration selection, and power down through a chain of shift registers distributed across the chip.

## 5. Device Fabrication

**Figure 7:**
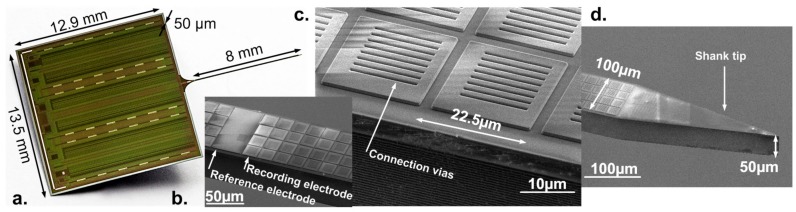
(**a**) Chip microphotograph showing complete probe with base and shank; (**b**) shank detail of small 20 µm × 20 µm and reference 40 µm × 80 µm electrodes; (**c**) detail of electrodes showing titanium nitride vias connecting the electrode to the internal metals and (**d**) details of the sharp shank tip and dimensions.

Figure 7a shows the chip photograph and details of the shank and electrodes after fabrication. The probes were fabricated using a 6M1P 0.13 µm Al CMOS technology and a 200-mm fab-compatible post-CMOS process is used for electrode deposition. The shank is 9 mm long, including a 1 mm neck, and 100 µm wide. A reliable shank thickness of 50 ± 3 µm and low bending of <100 µm were achieved by combining Si_3_N_4_ stress compensation with wafer backside thinning and polishing. The front side deep Si etch process defining the shank outline was optimized to achieve very smooth shank etch walls for minimal damage during implantation in rodent brain. The tip has a length of 300 µm and a sharp opening angle of 20°, a geometry targeting low tissue damage [24]. The dimensions of the probe base are 11.9 mm × 13.5 mm (width × height) and a thickness of 50 µm (Figure 7a), that is the same as the shank which is achieved through full wafer thinning.

**Figure 8:**
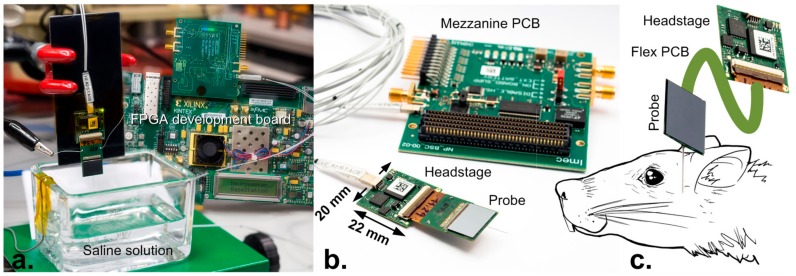
(**a**) Probe testing in saline solution attached to headstage, showing the back-end FPGA board in the background; (**b**) detailed view of probe and headstage connected to the mezzanine PCB (printed circuit board) through the flexible dual micro coaxial cable; the mezzanine board allows for connection of external battery for low noise and digital synchronization signals; (**c**) schematic display of probe implanted into animal.

To achieve the low-impedance and biocompatible TiN electrodes, a scalable and CMOS-compatible process was used. The 20 µm × 20 µm electrodes are arranged in a 4 × 336 array, with periodic interruptions for 12 large 20 µm × 80 µm reference electrodes (Figure 7b). Such a uniform arrangement of small electrodes covering the full shank allows for the capturing of spikes of single neurons with high spatial resolution. The center-to-center distances of the small neighboring sites is 22.5 µm, as shown in Figure 7c. If the electrode pitch is maintained, mask changes can allow for smaller or different shapes of electrodes. The large reference electrodes are not a requirement; however, they were purposely designed based on the neuroscientists’ recommendation. Larger sites will average the spikes around the reference, improving the recording quality. Multiple vias are used to connect the electrodes to the top CMOS metal line, which results in an increase in surface area and thus a reduction in the electrode impedance. The average electrode impedance for the 20 µm × 20 µm sites measured at 1 kHz in phosphate-buffered saline (PBS) of pH 7.4 was 48.1 ± 2.5 kΩ. After post-CMOS processing, the final probes are wire-bonded onto custom PCBs (Figure 8b). The probe base was covered by a metal-coated Si spacer that acts as a light-shield and reference surface during implantation. The bond-wires are finally sealed in a black bio-compatible epoxy (Master Bond EP42HT-2MED, Hackensack, NJ 0761, USA).

## 6. System

Due to the design constraints described in Section 2.2 and Section 2.3 regarding the chip dimensions and power dissipation in close proximity to the brain, certain functions need to be pushed off-chip. As a result, auxiliary circuitry is present on a small PCB (printed circuit board), called a headstage (Figure 8b), which is placed in the vicinity of the neural probe. 

The probe is wire bonded directly on a short and thin PCB, which attaches to the headstage through a zero insertion force (ZIF) connector (Figure 8b). The size of this short PCB is adaptable to the application and may be made flexible.

The small 20 mm × 22 mm headstage weighs 1.25 g and connects to a back-end FPGA development board through a 3 m, flexible, dual micro-coax cable. The cable is selected for maximum flexibility and low weight (3.5 g/m) to minimize the strain in freely moving animal experiments.

To provide a reliable and high speed data link between the headstage and back-end, a dedicated gigabit multimedia serial link serializer IC (MAX9271) and its corresponding de-serializer (MAX9272A) are used. The pair of ICs provide high speed data link with low power consumption and include error correction and detection codes for a high reliability data path.

Data communication between the serializer and deserializer is provided through a high bandwidth, unidirectional connection used for streaming the neural data, as well as a low bandwidth, bi-directional serial link used for the controlling and the configuration of the neural probe. Both connections are carried out across the same coaxial cable by the serializer and de-serializer pair. 

At the probe end, the headstage contains a small FPGA that is used for managing the neural probe configuration as well as generating the clock and analog calibration signals through an external digital-to-analog converter (DAC). The presence of a DAC on the headstage gives the neuroscientists the flexibility to envision other usages for the calibration or impedance measurement circuits. 

The headstage and neural probe are powered using the second micro coaxial cable. Multiple low noise, low drop voltage regulators are used to generate the required power rails on the headstage. 

At the back end, the system uses an off the shelf Xilinx Kintex 7 FPGA development board with an attached mezzanine PCB containing the de-serializer IC (Figure 8a,b).

A Gigabit Ethernet connection is used between the system and a PC to stream the data and control the probe. This connection allows for an increased distance to recording equipment, ground separation, as well as data splitting (i.e., sending data to multiple computers).

The FPGA development board provides 27 s of data buffering using the onboard RAM as well as preprocessing (including real time gain calibration). Additionally, 16 external digital signals are recorded simultaneously with the neural data to allow for synchronization with various external equipment. Furthermore, sufficient resources for closed loop neuroscience experiments are left available at the user’s disposal on the back-end FPGA development board.

## 7. Test Results

### 7.1. Electrical Performance

Measurements were performed in a dark Faraday cage, using phosphate buffered saline solution to contact the electrodes (Figure 8a). The total power consumption is 31 mW for 678 channels, with 2.3 mW dissipated in the shank (3 µW/PA), and 28.7 mW in the base, including data transmission with 4 pF loading.

**Figure 9:**
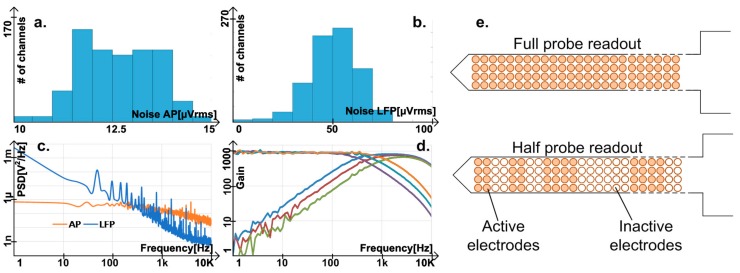
Adapted from [5]. Measurement results in half-probe readout, omitting the small number of defective channels; (**a**) distribution of noise in AP and (**b**) LFP band; (**c**) noise density in AP band (300 Hz–7.5 kHz) and LFP band (1 Hz–1 kHz); (**d**) different filter corner configurations, considering a fixed total gain of 1000; LFP high pass corners is below 1 Hz and not visible; (**e**) full probe readout and half probe readout allowing 6 random regions out of 12 to be active.

In half-probe recording mode (678 channels), the total input referred noise, including the electrodes, and using the broadest band is 12.4 ± 0.9 µVrms in the AP band (300 Hz–7.5 kHz) and 50.2 ± 12 µVrms in the LFP band (1 Hz–1 kHz), as shown in Figure 9. A reduction of LFP noise is possible by software averaging multiple channels, as LFP signals have low spatial resolution. In full-probe recording, 1356 channels can be simultaneously turned on for lower fidelity recording purposes, in which case the noise may increase up to 2.5 times, as explained in Section 4.4. 

The crosstalk across the full signal chain is −63 dB at 1 kHz, with the measurement being limited by the noise floor. 

Table 1 compares this work with prominent passive and active neural probes, showing up to a 3.5 times increase in the total number of channels compared to the state of the art, while maintaining similar performance when using half-probe recording.

### 7.2. In-Vivo Neural Recordings

We performed in vivo recordings in the brain of anesthetized rats to validate the CMOS probes. All of the animal experiments were performed according to the EC Council Directive of 24 November 1986 (86/89/EEC) and all procedures were reviewed and approved by the local ethical committee and the Hungarian Central Agricultural Office (license number: PEI/001/695-9/2015).

**Figure 10:**
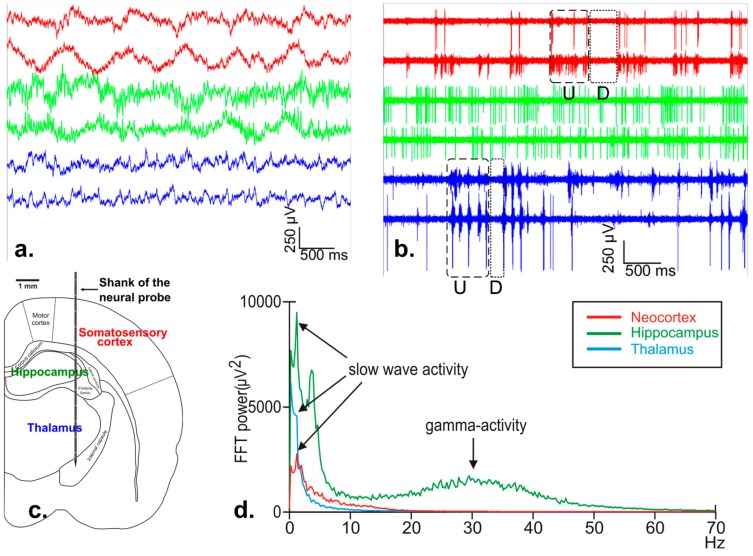
(**a**) local field potentials (LFP) simultaneously recorded from the neocortex (red), hippocampus (green), and thalamus (blue). Traces were obtained from the raw data recorded in LFP mode (internal reference, gain 500, low-pass 500 Hz); (**b**) multi- and single-unit activity recorded simultaneously from the neocortex (red), hippocampus (green), and thalamus (blue). Traces were recorded in action potentials (AP) mode (internal reference, gain 1000, high-pass 500 Hz). Dashed and dotted box indicate neocortical/thalamic up-states (U) and down-states (D); (**c**) schematic of a coronal rat brain section indicating the estimated position of neural recordings, d. fast Fourier Transform (FFT) plot of the recorded neural activity showing the dominant brain rhythms in the investigated brain areas during ketamine/xylazine anesthesia. Note that slow wave activity (1–1.5 Hz) appeared in all three brain structures, while high gamma activity (30–40 Hz) was present only in the hippocampus.

For the acute experiments, Wistar rats (*n* = 5, body weight: 270–450 g, gender balanced) were anesthetized with an intramuscular injection of ketamine/xylazine (KX) mixture (37.5 mg/mL ketamine and 5 mg/mL xylazine at 0.2 mL/100 g body weight injection volume). A craniotomy with an area of 3 × 3 mm^2^ was drilled over the left hemisphere, then a small piece of the dura mater was removed above the target site (anterior-posterior: −2.5 mm; medial-lateral: 3 mm, with reference to bregma; Figure 10c, [27]). Before insertion, the probe was connected to the headstage, which was mounted to a stereotaxic micromanipulator (David Kopf Instruments, Tujunga, CA, USA). After that, the CMOS probe was driven into the brain tissue to a depth of 6.5–7.5 mm either manually (insertion rate: ~0.1 mm/s, *n* = 3 insertions) or using a motorized stereotaxic device (Neurostar GmbH, Tübingen, Germany) with slow insertion rate (~2 μm/s, *n* = 2 insertions). The targeted brain areas were the trunk region of the somatosensory cortex and the underlying hippocampal and thalamic areas (Figure 10c). In the latter area, we could record the activity simultaneously from various thalamic nuclei (e.g., nucleus reticularis thalami, ventrobasal complex). A stainless steel needle inserted in the nuchal muscle of the animal served as the external reference electrode during the recordings.

The hardware and software components of the electrophysiological recording system and the CMOS probe have been tested successfully; spontaneous local field potentials (LFP), multi- and single-unit activity (MUA and SUA, respectively) could be recorded from neocortical, hippocampal and thalamic locations of the rat brain (Figure 10a,b,d). 

Furthermore, this provides an initial confirmation for the thermal model described in Section 2.3, as no degradation due to overheating was observed. However, since these recordings were short term, a more thorough evaluation through signal quality monitoring over longer experiments is needed. 

A brain rhythm, the so called slow wave activity (SWA), with a characteristic peak frequency of about 1 Hz that emerges in the thalamocortical system of rats during KX anesthesia (e.g., Figure 10a,d) was used as a benchmark to verify the recorded brain signals [28]. During SWA, the rhythmic alternation of two phases, both with a duration of a few hundred milliseconds can be observed in the neocortex and in various thalamic nuclei: up-states with high spiking activity and down-states with ceased action potential (AP) firing [28]. These two states could be clearly recognized on the cortical and thalamic recordings acquired in AP mode from the brain tissue of the anesthetized rats (Figure 10b). Furthermore, the neocortical depth profile of the SWA constructed from LFP and MUA traces was found to be comparable to our previous findings obtained with a laminar 24-channel passive silicon probe in the somatosensory cortex of KX-anesthetized rats [29]. In the hippocampus, beside the SWA, another dominant brain oscillation can be detected during KX-induced anesthesia: 30–40 Hz gamma activity [30]. This KX-induced hippocampal gamma activity is indicated in the power spectrum (computed from a hippocampal trace recorded in LFP mode) by an increased spectral power in the frequency range of 20–40 Hz (Figure 10d). 

**Figure 11:**
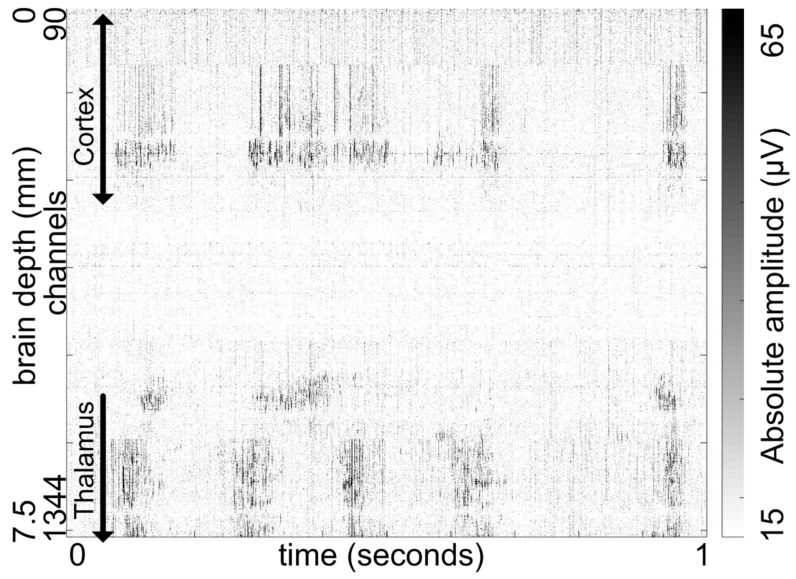
Representative spiking activity across more than 1250 channels of the probe shank, spanning approximately 7.5 mm of brain tissue. The raw data is shown. The spike-map was constructed from 1 second of data recorded in AP mode; the time series of each channel’s data is plotted as a horizontal line using brightness to encode the absolute amplitude, with darker areas being an indication of neural spiking activity. Ketamine/xylazine anesthesia induces slow wave activity (with a peak frequency of 1–1.5 Hz) or delta rhythm (1.5–4 Hz) in the neocortex and thalamus, which can be observed as a rhythmic alternation of high and low spiking activity. Notes: the first ~90 channels are not displayed as they were outside of the brain and only recorded noise; the picture requires one line per channel (~1250), therefore resolution of the provided image was scaled down. Occasionally neurons near the reference electrode may spike, causing a line to be displayed on all channels using that specific local reference. Such artefacts can be eliminated during offline processing.

By using full probe recording, we were able to record the brain electrical activity from more than 1250 electrodes simultaneously (Figure 11). This allowed us to monitor the spiking activity during SWA in the neocortex and in various nuclei of the thalamus at the same time, with both high spatial and temporal resolution. The SWA, which is thought to be generated in the thalamocortical network, has a complex spatiotemporal dynamic with the underlying mechanisms still barely known due to the lack of appropriate apparatuses to record brain activity from multiple, large areas of the neocortex and thalamus simultaneously. Therefore, the use of high-channel count, high-density neural probes might have a great potential to significantly further our knowledge of the SWA in the near future. 

One of the fundamental analysis methods in the field of neuroscience is the examination of the spiking activity of individual neurons and correlating their activity to different brain states, external stimuli, or certain behaviors. Since the mammalian brain contains millions of neurons, it is essential to record the simultaneous activity of as many neurons as possible. State-of-the-art silicon-based probes can monitor the spikes of several dozen to a few hundred neurons at once [31,32,33]. To assess the single unit yield of the CMOS probe, we performed spike sorting on the data recorded in AP mode using a software capable to process high-channel-count recordings [34]. Full probe recordings obtained from three of five rats were analyzed. In total, 247 well-separable single units were sorted from the neocortex (mean ± standard deviation (SD) of neuron clusters, 29.67 ± 10.5; range, 19–40) and the thalamus (52.67 ± 21.39, 34–76). The peak-to-peak amplitude of the mean spike waveform of these units usually exceeded 100 μV suggesting good separation from other neuron clusters and the background activity (Neocortex, mean ± SD, 293.23 ± 138.31 μV, range, 96–786 μV; Thalamus, 268.86 ± 117.98 μV, 100–661 μV). Using a less conservative sorting approach (including units with spike amplitudes below 100 µV, but still with clear refractory periods on their auto correlograms) would yield additional two dozen neuron clusters in both structures. Therefore, by using full probe recording, the activity of about a hundred or more neurons can be monitored with a single CMOS probe simultaneously. Using multiple probes in the same animal at the same time might further increase the unit yield. However, it is important to note that several factors may influence the number of separable single units, e.g., the actual brain state, the investigated brain areas, the spike sorting method used, or the tissue damage caused during probe implantation. Furthermore, we used relatively short recordings (~5 min) for spike sorting, therefore a significant amount of neurons with low firing rates might have been omitted. Hence, the single unit yield provided here is rather an underestimation of the actual unit number.

**Figure 12:**
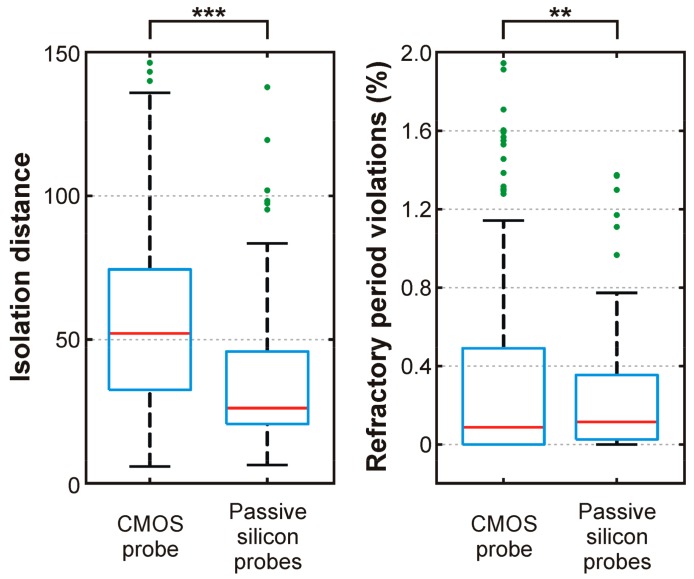
Cluster quality metrics (isolation distance and refractory period violations) calculated for single units recorded with the CMOS probe (*n* = 247) and with passive silicon probes (*n* = 101). Red line: median; blue box: 1st quartile–3rd quartile; whiskers: 1.5× interquartile range above and below the box; green dots: outliers. Extreme outliers are not displayed (isolation distance: 12 data points from the CMOS probe data ranging from 183 to 475; refractory period violations: 22 data points from the CMOS probe data ranging from 2.2 to 13.1 percent and 3 data points from the passive silicon probe data ranging from 2.8 to 4.2 percent). **: *p* < 0.01; ***: *p* < 0.001.

To quantitatively assess the quality of the isolated neuron clusters, we calculated two measures commonly used for this purpose: the isolation distance and the percentage of spikes violating the absolute refractory period (<2 ms) of neurons (Figure 12, [35,36]). Furthermore, we also computed these measures for neuron clusters (*n* = 101) obtained from data recorded with passive silicon probes (laminar (A1x32-6mm-50-177) or Buzsaki64 type probes from NeuroNexus Technologies) from the somatosensory cortex of rats. Four 10-min-long recording files were analyzed, which were acquired either under ketamine/xylazine anesthesia (*n* = 3) or under urethane anesthesia (*n* = 1). Isolation distance values of single units recorded with the CMOS probe were found significantly higher as compared to the isolation distance values of neuron clusters recorded with traditional silicon probes (*p* < 0.001, Student’s *t*-test). Furthermore, although the difference between the active and passive probe data was significant in terms of the second measure as well (*p* < 0.01, Student’s *t*-test), most of the neuron clusters had refractory period violations below 1%, suggesting that the majority of clusters contained only a low number of spikes fired by other neurons. These results suggest that the CMOS probe is capable of recording single unit activity with a quality as good as, or even better than, traditional silicon probes.

**Figure 13:**
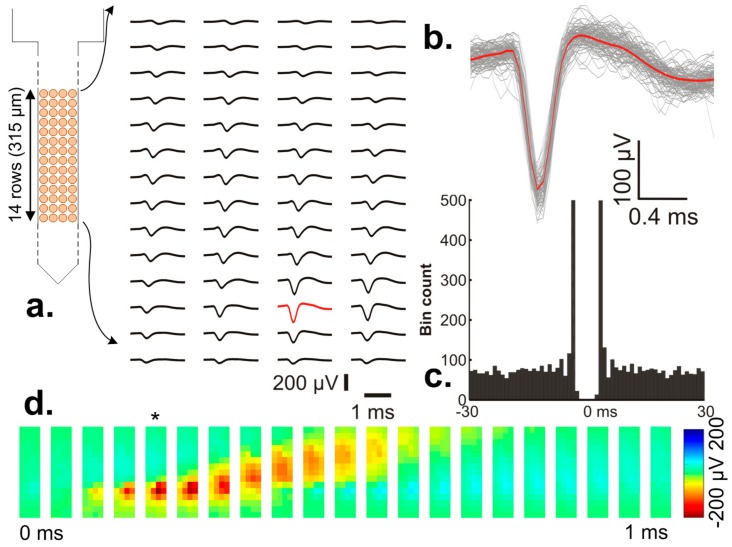
(**a**) The mean spike waveforms of a putative neocortical pyramidal cell captured on 4 × 14 electrodes. The waveform with the largest peak-to-peak amplitude is colored red; (**b**) individual spikes (waveforms in gray color, *n* = 90) of the same pyramidal cell recorded by the electrode corresponding to the red waveform in panel a. The mean spike waveform is displayed in red color; (**c**) the autocorrelogram of the demonstrated pyramidal neuron (bin size: 1 ms). The two peaks indicate burst firing (multiple spikes fired in rapid succession); (**d**) Color-coded potential distribution maps corresponding to different time points of the mean spike waveform. The maps are visualized according to the layout of the 4 × 14 electrodes. The potential map corresponding to the time point of the negative peak of the mean spike waveform shown in panel b is indicated with an asterisk. Note the temporal propagation of the negative peak of the spike (red patch) from lower electrodes to upper electrodes. The spikes of the neuron were recorded in AP mode (internal reference, gain 500, high-pass 500 Hz).

The high spatial resolution of the probe allows for spikes of the same neuron to be recorded on multiple, adjacent electrodes, providing a two-dimensional map of the neuron’s spike waveform with both high spatial and temporal resolution (Figure 13). The mean spike waveforms of a putative pyramidal cell calculated from the sorted spikes of the single unit recorded on 4 × 14 electrodes are shown in Figure 13a. Individual spikes of the isolated neuron cluster recorded on a single electrode (Figure 13b) and its autocorrelogram (Figure 13c) indicate good unit separation quality. Based on color-coded maps constructed from the two-dimensional mean spike waveform of the neuron (Figure 13d), the backpropagation of the AP into the apical dendritic shaft (propagation of the red patch in Figure 10d that corresponds to the negative peak of the spike waveform) could be observed during the time course of the action potential, a phenomenon typical of pyramidal cells [37]. Furthermore, probes produce similar data as recorded in a more traditional way of using passive silicon probes, with an analogous layout of recording sites [38,39] and external amplifiers. In conclusion, our results suggest that the CMOS probe system may provide valuable neural data from multiple brain sites of rodents with high spatial resolution. High-resolution electrical images of action potentials provided by these probes allow for the detailed examination of the spatiotemporal dynamics of spikes recorded in vivo or in the near future may be applied to identify various types of neocortical neurons.

## 8. Conclusions

Attempting to multiplex active electrodes on a long and narrow shank in order to increase the number of simultaneous readout channels comes with a series of drawbacks and limitations. By implementing various innovative circuit design techniques (required to mitigate power supply drop and ripple, bias generation, filter and amplifier instability, as well as noise folding) we have succeeded in designing a new type of neural amplifier. With the help of this amplifier, we have demonstrated the first high density, multiplexed active neural probe capable of recording the complete set of electrodes present on the shank.

As such, this work demonstrates an active neural probe featuring 1356 simultaneous recording channels that are equivalent to a 3.5 times increase when compared to the state of the art. Extensive in vivo probe validation has been carried out to demonstrate the expected capabilities of the device. 

By providing the possibility to record the entire length of the shank as well as providing high density and increased electrode count, this novel active neural probe opens the possibility of new types of neuroscience observations, as demonstrated briefly in the captured in vivo data.

## Figures and Tables

**Table 1:** Comparison with prior art.

Parameter	Measured Values							
	[3]	[13]	[25]	[9]	[26]	[2]	[4]	This Work
**Probe Shank**								
No. Electrodes	64	64	--	334	--	455	966	**1356**
Electrode Pitch [μm]	100	24	--	30	--	35	20	**22.5**
CSAC [μm^2^]	127.5	30.55	--	11.98	--	10.99	3.65	**3.7**
Total Power/El [μW]	--	--	--	--	--	3.6	4.7	**3**
Crosstalk [dB]	--	-84	--	--	--	−44.8	−64	**−63**
**Probe Base (Recording System)**								
No. recording channels with specified noise	8	64	100	16	96	52	384	**678**
Max no. of channels	8	64	100	16	96	52	384	**1356**
Gain	1000	194	400/600	--		30–4000	50–2500	**50–2500**
HP Corner [Hz]	300	1.3	0.25	--	300	0.5/200/ 300/500	0.5/300/ 500/1000	**0.5/300/** **500/1000**
LP Corner [Hz]	10,000	6400	2500–10,000	--	10,000	200/ 6000	1000/ 10,000	**300/500/** **1000/8000**
ADC Resolution [b]	5	--	9	--	10	10	10	**10**
Sampling Rate [kS/s]	160 (8 Ch)	--	200 (10 Ch)	--	31/ Ch	120 (4 Ch)	390 (13 Ch)	**400** **(20 Ch)**
**Full probe**								
Total Power/Ch [μW]	94.5	351.6	0.94 *	--	67	27.84	49	**45**
Total Area/Ch [mm^2^]	0.625	0.45	0.25	--	0.26	0.19	0.12	**0.12**
Input Referred Noise AP band [μVrms]	9.2 ^#^	1.7 ^#^	3.2 ^#^	--	2.2^#^	3.2	6.36	**12.4**
Input Referred Noise LFP band [μVrms]	--	2 ^#^	3.8 ^#^	--	--	5.8	10.3	**50.2**

^#^ Circuit noise only, excludes electrode noise. * IO digital power not included in this number.
